# Progress Toward Understanding Infection-Associated Chronic Conditions and Illnesses

**DOI:** 10.3201/eid3114.251187

**Published:** 2025-12

**Authors:** Anthony E. Fiore

**Affiliations:** Centers for Disease Control and Prevention, Atlanta, Georgia, USA (retired); Emerging Infectious Diseases, Centers for Disease Control and Prevention, Atlanta

**Keywords:** Infection-associated chronic conditions and infections, IACCI, chronic illness, Long COVID, chronic fatigue syndrome, myalgia encephalomyelitis, COVID-19, viruses, bacteria

Infection-associated chronic conditions and illnesses (IACCIs) are a variety of health consequences that occur after an acute infection (*1*). Chronic sequealae considered IACCIs include various combinations of infection-associated organ damage, autoimmune conditions, and persistent unexplained systemic symptoms, such as debilitating fatigue, postexertional malaise, cognitive impairment, musculoskeletal pain, and sleep disorders (*1*). The health and societal impact of long COVID over the past 5 years, and recognition of long COVID as an IACCI, has reinvigorated the study of these poorly understood disorders (*2*–*7*).

This supplement of *Emerging Infectious Diseases* features a series of studies undertaken by the Centers for Disease Control and Prevention to increase awareness and understanding of IACCIs and to highlight available prevention and treatment resources for public health practitioners, healthcare providers, and the public. The supplement includes research regarding IACCIs occurring after infections caused by SARS-CoV-2 (COVID-19), respiratory syncytial virus, *Giardia lamblia*, *Coccidioides immitis*, *Borrelia burgdorferi* and other *Borrelia* species (Lyme disease), and West Nile virus. The studies describe various approaches to characterizing IACCIs, which include measuring prevalence, persistence, management, and duration of a defined syndrome; assessing absenteeism attributable to an IACCI; and estimating risk for life-threatening sequalae, such as increased thrombotic events.

IACCIs can emerge with a heterogenous range of signs, symptoms, and laboratory results and are not consistent in duration, presentation, or severity, even when grouped according to the known or suspected previous infection, suggesting that multiple underlying mechanisms could be responsible for illness (*1*,*2*). Suspected mechanisms include continued immune stimulation from antigens or continued infection in a sequestered body site that cannot be sampled, reactivation of latent viruses, autoimmune responses, microbiome dysbiosis, persistent tissue damage, disordered coagulation, and disrupted nerve signaling (*1*,*2*). IACCIs cause marked disruption to a patient’s ability to return to work or school or to resume their life as it was before the inciting infection. Complicating the frustrated patient’s predicament, clinicians attempting unproven treatments (e.g., repeated antibiotic courses) might exacerbate illness by introducing additional risks or temporarily masking potentially treatable causes.

The medical community now has a framework for how research on IACCIs should be developed. In 2024, the National Academies of Science, Engineering, and Medicine (NASEM) proposed a definition to harmonize terminology and measurement approaches and to formulate a research agenda for “infection-associated chronic illnesses” (authors in this supplement have used the term IACCI to also include health conditions that also are debilitating) (*2*). In terms of IACCIs attributable to COVID-19, NASEM proposed a long COVID definition, and the American Academy of Physical Medicine and Rehabilitation established the Multi-Disciplinary Post-Acute Sequelae of SARS-CoV-2 Infection Collaborative to develop multidisciplinary guidance on clinical management (*8*). Of note, many of the IACCIs discussed in this supplement have similarities to or include myalgia encephalomyelitis and chronic fatigue syndrome in their definition. Although questions remain regarding the pathophysiology of each, adapting clinical management of such syndromes to IACCIs may be useful (*9*,*10*).

Patients suffering with IACCIs often receive misdiagnoses or become stigmatized, both of which impede potentially beneficial clinical management strategies. Without an understanding of pathophysiology, diagnosis, treatment, and care for patients with IACCIs, healthcare providers will continue struggling to provide patients with accurate diagnoses and potentially effective treatment and support. This supplement aims to provide additional insights into several IACCIs and focus more attention on these poorly understood conditions.

## References

[1] Choutka J, Jansari V, Hornig M, Iwasaki A. Unexplained post-acute infection syndromes. Nat Med. 2022;28:911–23. 10.1038/s41591-022-01810-6 35585196

[2] National Academies of Sciences, Engineering, and Medicine; Health and Medicine Division; Board on Health Sciences Policy; Board on Global Health; Forum on Neuroscience and Nervous System Disorders; Forum on Microbial Threats. Toward a common research agenda in infection-associated chronic illnesses: proceedings of a workshop. Snair M, Liao J, Ashby E, Biffl C, editors. Washington: National Academies Press [cited 2025 Aug 5]. 10.17226/27462 38648305

[3] National Academies of Sciences, Engineering, and Medicine; Health and Medicine Division; Board on Global Health; Board on Health Sciences Policy; Committee on Examining the Working Definition for Long COVID. A long COVID definition: a chronic, systemic disease state with profound consequences. Goldowitz I, Worku T, Brown L, Fineberg HV, editors. Washington: National Academies Press [cited 2025 Aug 5]. 10.17226/27768 39110819

[4] Ewing AG, Salamon S, Pretorius E, Joffe D, Fox G, Bilodeau S, et al. Review of organ damage from COVID and Long COVID: a disease with a spectrum of pathology. Med Rev (2021). 2024;5:66–75. 10.1515/mr-2024-0030 39974559 PMC11834749

[5] Vahratian A, Saydah S, Bertolli J, Unger ER, Gregory CO. Prevalence of post-COVID-19 and activity-limiting post-COVID-19 condition among adults. JAMA Netw Open. 2024;7:e2451151. 10.1001/jamanetworkopen.2024.51151 39671200 PMC11645641

[6] Perlis RH, Lunz Trujillo K, Safarpour A, Santillana M, Ognyanova K, Druckman J, et al. Association of post–COVID-19 condition symptoms and employment status. JAMA Netw Open. 2023;6:e2256152. 10.1001/jamanetworkopen.2022.56152 36790806 PMC9932847

[7] Department of Health and Human Services, Office of the Assistant Secretary for Health. National Research Action Plan on Long COVID. 2022 [cited 2025 Sept 4]. https://www.autoimmuneinstitute.org/wp-content/uploads/2025/03/The-National-Research-Action-Plan-on-Long-COVID.pdf

[8] Cheng AL, Herman E, Abramoff B, Anderson JR, Azola A, Baratta JM, et al. Multidisciplinary collaborative guidance on the assessment and treatment of patients with Long COVID: A compendium statement. PM R. 2025;17:684–708. 10.1002/pmrj.13397 40261198 PMC12162235

[9] Unger ER, Lin JS, Brimmer DJ, Lapp CW, Komaroff AL, Nath A, et al. CDC Grand Rounds: chronic fatigue syndrome—advancing research and clinical education. MMWR Morb Mortal Wkly Rep. 2016;65:1434–8. 10.15585/mmwr.mm655051a4 28033311

[10] Centers for Disease Control and Prevention. Myalgic encephalomyelitis/chronic fatigue syndrome healthcare provider toolkit, May 13, 2024 [cited 2025 Aug 5] https://www.cdc.gov/me-cfs/hcp/toolkit/index.html

